# Cellular and molecular evidence for a role of tumor necrosis factor alpha in the ovulatory mechanism of trout

**DOI:** 10.1186/1477-7827-8-34

**Published:** 2010-04-12

**Authors:** Diego Crespo, Emilie Bonnet, Nerea Roher, Simon A MacKenzie, Aleksei Krasnov, Frederick W Goetz, Julien Bobe, Josep V Planas

**Affiliations:** 1 Departament de Fisiologia, Facultat de Biologia, Universitat de Barcelona and Institut de Biomedicina de la Universitat de Barcelona (IBUB), 08028 Barcelona, Spain; 2 Institut National de la Recherche Agronomique (INRA), UR1037 SCRIBE, IFR140, Campus de Beaulieu, F-35000 Rennes, France; 3 Unitat de Fisiologia Animal, Departament de Biologia Cellular, Fisiologia i d'Immunologia, Facultat de Ciències, Universitat Autònoma de Barcelona (UAB), Spain; 4 Nofima Akvaforsk Fiskeriforskning AS, PO Box 5010, Ås NO-1430, Norway; 5 Great Lakes Water Institute, University of Wisconsin-Milwaukee, Wisconsin, USA

## Abstract

**Background:**

The relevance of immune-endocrine interactions to the regulation of ovarian function in teleosts is virtually unexplored. As part of the innate immune response during infection, a number of cytokines such as tumor necrosis factor alpha (TNF alpha) and other immune factors, are produced and act on the reproductive system. However, TNF alpha is also an important physiological player in the ovulatory process in mammals. In the present study, we have examined for the first time the effects of TNF alpha in vitro in preovulatory ovarian follicles of a teleost fish, the brown trout (*Salmo trutta*).

**Methods:**

To determine the in vivo regulation of TNF alpha expression in the ovary, preovulatory brook trout (Salvelinus fontinalis) were injected intraperitoneally with either saline or bacterial lipopolysaccharide (LPS). In control and recombinant trout TNF alpha (rtTNF alpha)-treated brown trout granulosa cells, we examined the percentage of apoptosis by flow cytometry analysis and cell viability by propidium iodide (PI) staining. Furthermore, we determined the in vitro effects of rtTNF alpha on follicle contraction and testosterone production in preovulatory brown trout ovarian follicles. In addition, we analyzed the gene expression profiles of control and rtTNF alpha-treated ovarian tissue by microarray and real-time PCR (qPCR) analyses.

**Results:**

LPS administration in vivo causes a significant induction of the ovarian expression of TNF alpha. Treatment with rtTNF alpha induces granulosa cell apoptosis, decreases granulosa cell viability and stimulates the expression of genes known to be involved in the normal ovulatory process in trout. In addition, rtTNF alpha causes a significant increase in follicle contraction and testosterone production. Also, using a salmonid-specific microarray platform (SFA2.0 immunochip) we observed that rtTNF alpha induces the expression of genes known to be involved in inflammation, proteolysis and tissue remodeling. Furthermore, the expression of kallikrein, TOP-2, serine protease 23 and ADAM 22, genes that have been postulated to be involved in proteolytic and tissue remodeling processes during ovulation in trout, increases in follicles incubated in the presence of rtTNF alpha.

**Conclusions:**

In view of these results, we propose that TNF alpha could have an important role in the biomechanics of follicle weakening, ovarian rupture and oocyte expulsion during ovulation in trout, primarily through its stimulation of follicular cell apoptosis and the expression of genes involved in follicle wall proteolysis and contraction.

## Background

Fish are exposed to a variety of pathogens and stressful conditions which may cause severe reproductive consequences [1,2]. It is now known that activation of the immune system as a result of a bacterial infection is characterized by the production of a wide variety of immune factors, including pro-inflammatory cytokines and chemokines [3] which can act as local chemical modulators or be secreted into the circulation and act as hormones. In particular, exposure to lipopolysaccharide (LPS), an active component of the wall of Gram-negative bacteria, induces the expression of TNFα in trout macrophages [4]. Once secreted into the circulation, the pleiotropic cytokine TNFα can then act on a number of different tissues, including non-immune tissues.

In mammals, TNFα is known to act in the ovary, where it affects differentiation, proliferation, steroid production, inflammation and induction of apoptosis through specific receptors [5-7]. Interestingly, TNFα is considered to be a mediator of ovulation through its stimulatory role on follicular apoptosis and breakdown of the extracellular matrix (ECM) in the follicle wall [8,9]. Therefore, in addition to its local production and action, TNFα can act as a mediator signaling the status of the immune system to the reproductive system. In fish, very little is known about the interaction between the immune and the reproductive systems and, in particular, of the involvement of TNFα in the reproductive process. We have previously reported that LPS administration results in the induction of apoptosis in the trout ovary and that preovulatory trout follicles incubated with conditioned medium from LPS-stimulated trout macrophages showed an increase in follicle contraction, suggesting that factors produced by trout macrophages in response to LPS may stimulate follicle contraction [10]. In view of these observations, we hypothesize that TNFα could be one of the most likely mediators of the effects of LPS in the trout ovary. Therefore, it is important to address the issue of the impact of the activation of the immune response by TNFα on reproductive function in fish.

In the present study, we have examined the effects (*in vitro*) of recombinant trout TNFα (rtTNFα) on the reproductive function of preovulatory brown trout (*Salmo trutta*) at various levels: (1) ovarian apoptosis and granulosa cell viability, (2) follicle contraction and steroid production and (3) gene expression profiles (microarray and qPCR analyses).

## Methods

### Animals

Reproductively mature female brown trout (*Salmo trutta*) from a cultured stock at the Piscifactoria de Bagà (Generalitat de Catalunya, Bagà, Spain) were kept under natural conditions of temperature and photoperiod. Fish at the preovulatory stage (according to the position of the germinal vesicle (GV) in oocytes that were cleared using a solution previously described [11]), were anesthesized in 3-aminobenzoic acid ethyl ester (0.1 g/l; Sigma, Alcobendas, Spain) dissolved in fresh water, and the fish were sacrificed by concussion prior to the collection of the ovaries. The dissected ovaries were immediately used for the various *in vitro *assays. For the experiment on the *in vivo *regulation of TNFα expression in the ovary, preovulatory brook trout (*Salvelinus fontinalis*) were briefly anesthesized in 3-aminobenzoic acid ethyl ester (0.1 g/l) and injected intraperitoneally with either saline (n = 3) or *E. coli *lipopolysaccharide (LPS) (3 mg/kg weight; Sigma) (n = 3) once a day over four consecutive days, as previously reported [10]. Twenty four hours after the last injection, fish were anesthesized (as described above) and sacrificed by spinal transection prior to the collection of the ovaries. Ovarian tissue (previously de-yolked by gentle pressure) was flash frozen in liquid nitrogen and stored at -80°C, until RNA isolation.

### Hormones and reagents

Coho salmon (*Oncorhynchus kisutch*) luteinizing hormone (sLH) was a kind gift from Dr. Penny Swanson (National Marine Fisheries Service, Seattle, WA) [12] and was dissolved directly in incubation medium.

For the generation of rtTNFα, the mature form of TNFα from brook trout (*Salvelinus fontinalis*) was subcloned into an expression vector, produced in *E. coli*, refolded, concentrated and shown to be biologically active using a fish cell line [13].

### Ovarian tissue incubations

After dissection, brown trout preovulatory ovaries were placed in Hank's balanced salt solution (HBSS) and individual ovarian follicles were manually separated with forceps from each ovary on ice, as previously described [14]. For the follicle contraction experiments, brown trout preovulatory follicles punctured using a 25-gauge hypodermic needle were incubated in HBSS containing 0.2% BSA (fraction V, Sigma; HBSS-BSA) in 6 cm culture dishes (10 follicles/4 ml) in the presence of the test compounds for 16 h at 15°C under shaking conditions (100 rpm). Follicle contraction was determined by measuring the weight of the 10 follicles in each replicate after incubation, as previously described and validated for epinephrine stimulation [15,16]. Since contraction results in the expulsion of yolk through the puncture site, a decrease in follicle weight indicates an increase in follicle contraction. For *in vitro *steroid production experiments, intact preovulatory brown trout follicles were incubated (10 follicles/well/1 ml) in HBSS-BSA in 24-well culture plates, in the absence or presence of different test compounds for 24 h at 15°C with gentle shaking (100 rpm). At the termination of the incubation, the medium was removed and stored at -20°C, until assayed.

To collect ovarian tissue for RNA extraction, preovulatory follicles from each of a total of three females were incubated (400 follicles/50 ml) in HBSS-BSA in the absence or presence of rtTNFα (100 ng/ml, dissolved directly in HBSS-BSA), at 15°C for 24 h with gentle shaking (100 rpm). At the end of the incubation follicles (previously de-yolked by gentle pressure) were removed, flash frozen in liquid nitrogen and stored at -80°C until assayed.

### Steroid radioimmunoassay

The concentration of testosterone (T) in brown trout ovarian follicle incubates were measured directly using commercial radioimmunoassay (Schering-CIS, Madrid, Spain), as described previously [17,18].

### Analysis of apoptosis and cell viability

Granulosa cell viability was assessed by incubating brown trout ovarian follicles in the absence or presence of 100 ng/ml rtTNFα for 18 h at 15°C, separating the granulosa layers and staining them with propidium iodide (PI; 50 μg/ml; Sigma). Subsequently, sheets of granulosa layers were mounted onto glass slides, counterstained with DAPI and visualized under a fluorescent microscope. To determine the incidence of apoptosis after rtTNFα treatment, granulosa cells were obtained from granulosa layers (isolated from control and rtTNFα-treated follicles) by enzymatic digestion with 0.2% collagenase type IA (*Clostridium histolyticum*; Sigma) under gentle agitation for 2 h at room temperature. After incubation, the homogenate was filtered through a 100 μm filter and subsequently through a 40 μm filter before centrifugation for 6 min at 500 g. The pellet was washed with 0.5 ml 1 × PBS and centrifuged 5 min at 500 g. Isolated granulosa cells were resuspended in 70% ethanol and stored at -20°C until use. To analyze the incidence of apoptosis, granulosa cells were resuspended in PI staining buffer (20 μg/ml PI, 0.1% Triton X-100, 200 μg/ml RNase A; Sigma) prior to analysis by flow cytometry (FACS analysis).

### RNA isolation and cDNA synthesis

Total RNA from ovarian tissue was isolated using TRIzol Reagent (Invitrogen, Barcelona, Spain), following the manufacturer's instructions and guidelines. Quantification was carried out with Qubit fluorometer (Invitrogen). cDNA synthesis was performed on 5 ìg DNase-treated (RQ1 DNase, Promega, Barcelona, Spain) total RNA using SuperScript III Transcriptase (Invitrogen), oligo(dT) primer and random hexamer primers (Promega), according to the manufacturer's protocols. The RNA was stored at -80°C until use.

### Microarray analyses

Microarray analyses were performed using the salmonid fish cDNA microarray platform (SFA2.0 immunochip) previously validated and described [19,20] that has been deposited in GEO under accession number GPL6154. Total RNA from pooled control and TNFα-treated brown trout preovulatory follicles (n = 20) from each of a total of three females was labelled with Cy3-dUTP and Cy5-dUTP (GE Healthcare, Barcelona, Spain) using SuperScript III Transcriptase. The cDNA synthesis reaction was performed at 50°C for 2 h in a 20 μl reaction volume, followed with RNA degradation with 0.2 M NaOH at 37°C for 15 min and alkaline neutralization with 0.6 M Hepes. Labeled cDNA was purified with Microcon YM30 (Millipore, Madrid, Spain). We used a dye swap experimental design and each pooled sample (20 follicles) from each female was hybridized to two microarrays. For the first slide, pooled test and control cDNA were labeled with Cy5 and Cy3 respectively, and for the second array dye assignment was reversed. The slides were pretreated with 1% BSA (fraction V), 5 × SSC, 0.1% SDS (30 min at 50°C) and washed with 2 × SSC (3 min) and 0.2 × SSC (3 min) at room temperature and hybridized overnight at 60°C in a cocktail containing 1.3 × Denhardt's, 3 × SSC, 0.3% SDS, 2.1 μg/μl polyadenylate and 1 μg/μl yeast tRNA. All chemicals were from Sigma. After hybridization, slides were washed at room temperature in 0.5 × SSC and 0.1% SDS for 15 min, 0.5 × SSC and 0.01% SDS for 15 min, and twice in 0.06 × SSC for 2 and 1 min, respectively. Scanning was performed with ScanArray 5000 (GSI Lumonics) and images were processed with GenePix Pro 5.0 (Axon). The measurements in spots were filtered by criteria *I/B *≥ 3 and (*I-B*)/(*S*_*I *_+ *S*_*B*_) ≥ 0.6, where *I *and *B *are the mean signal and background intensities and *S*_*I*_, *S*_*B *_are the standard deviations. After subtraction of median background from median signal intensities, the expression ratios (ER) were calculated. Locally weighted non-linear regression (Lowess) normalization was performed, first for the whole slide and next for twelve rows and four columns per slide. The differential expression was assessed by the difference of the mean log_2 _ER from zero between the slides with reverse labelling (6 spot replicates per gene on each slide, Student's t-test, p < 0.01). The log_2 _ER ranked up- or down-regulated genes were analyzed interrogating the functional classes of Gene Ontology (GO) [21] and compared by the sums of ranked genes (Student's t-test, p < 0.05). Complete microarray results were submitted to NCBI GEO Omnibus (GSE20296).

### Quantitative real-time PCR (qPCR)

In order to quantify mRNA expression of individual genes, quantitative real-time PCR (qPCR) was carried out with the same samples used for microarray analyses (pooled follicles from each of three separate females). cDNA was diluted 1:25 for target mRNA and 1:2000 for 18S and used as a template. The reactions (20 μl final volume) contained 10 μl of SYBR GreenER qPCR SuperMix (Invitrogen), 500 nM concentration of forward and reverse primers and 5 μl of cDNA. Reactions were run in a iCycler Thermal Cycler (BioRad, Alcobendas, Spain) under the following protocol: 2 min at 50°C, 8 min at 95°C, followed by 40 cycles of 15 sec denaturation at 95°C and 30 sec at 60°C, and a final melting curve of 81 cycles from 55°C to 95°C (0.5°C increments every 10 sec). All samples were run in triplicate and fluorescence was measured at the end of every extension step. Fluorescence readings were used to estimate the values for the threshold cycles (*C*t). Relative expression of mRNA was evaluated by -ΔΔ*C*t, based on the quantification method by Livak and Schmittgen [22]. As the reference gene, 18S ribosomal RNA was selected. For all primer pairs, a dilution curve obtained from a serially diluted ovarian cDNA pool was used to ensure that PCR efficiency was higher than 90%. Primer sequences, amplicon sizes and GenBank accession numbers of the target genes are presented in Table 1.

**Table 1:** Sequences of primers used in gene expression analyses by qPCR

Clone ID	Clone name	Primer sequence (5'-3')	Amplicon size (bp)
AJ278085	Tumor necrosis factor alpha (TNFα)	(F) AGCATGGAAGACCGTCAA (R) TTCGTTTACAGCCAGGCT	271
CA344136	Small inducible cytokine B14 precursor	(F) AGCTGAAGCCTACAAGTGCAGGTGC (R) TCCTTCCAGATCCGGAACCAC	219
CU068239	Leukocyte cell-derived chemotaxin 2	(F) AGGACACCACGGCGCAAGCAGAG (R) TGTCACACATCTGGACGTGGACG	314
CA374135	CCL4	(F) TGCTTGTTCTGGTACTCAGTGCCA (R) TGTCTGAGGACCTGGCACACACC	207
CA351440	TNF decoy receptor	(F) TCTCCTGGTATTTGCGCTCTGTGGT (R) TATAAGTCGGTGTGTGAGCGCCTGA	65
DW550559	Coronin-1C	(F) AGTGGTGCGAGCGAGTAAGT (R) GACAGGTCCCGTATGACCAC	241
CU067298	Triosephosphate isomerase	(F) GCTTCCTTGTGGGTGGTGCTTC (R) CCACAGGAAGGAGCAGATGACACC	181
CU069718	Serine protease-like protein-1	(F) ACCAAAAACATGCTGTGTGC (R) CCCTCCATTTGAAGTGATCC	111
CA364711	Inhibitor of apoptosis protein 3	(F) TGCTTCTGCTGTGGAGGGATG (R) CGTGGTCAATAGGGTGCTGGA	244
AF005026	Kallikrein (KT-14)	(F) CCAGGAGGTGGAATTAGGATGAGA (R) GCAGAGCAGCAGTCAGGAACA	167
AF223388	Novel osteopontin-like protein (NOP)	(F) CAGCAGAGAGCAACGAGAGCCAT (R) AGGGTCATAGAGCACCTTCCAGG	182
U67854	Trout ovulatory protein-2 (TOP-2)	(F) GGGATGTGTGCGGAGTTATGCT (R) ACTGTGTCGGTGCAATGCAGTCAT	99
AF156738	Trout decoy receptor (TDcR)	(F) GCGTCGGTTAGCAATGAGGC (R) GGTTTCTTGCCTCCCTCTTGTG	45
CA380218	Macrophage migration inhibitory factor (MIF)	(F) AGGCAACAGATGAACTGGCCAAGG (R) AGGCCACATTCGCTGCCTCCA	258
AF308735	18S ribosomal RNA	(F) CGGAGGTTCGAAGACGATCA (R) TCGCTAGTTGGCATCGTTTAT	62

F: forward, R: reverse.

### Statistical analysis

Differences among groups were analyzed for statistical significance with non parametric Mann-Whitney U test or by one-way analysis of variance (ANOVA) followed by the Fisher protected least significant different test, using StatView 5.0 (SAS Institute, Cary, NC). Results are expressed as means ± SE and differences between groups were considered to be significant if p < 0.05.

## Results

**Figure 1:**
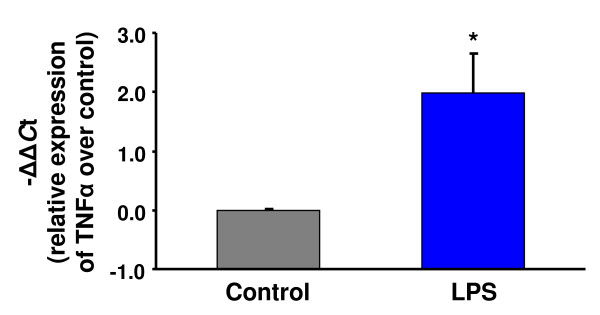
**Expression of tumor necrosis factor α (TNFα) in the brook trout ovary in response to lipopolysaccharide (LPS) administration *in vivo***. The relative expression of TNFα was determined by real-time PCR (qPCR) and normalized to the abundance of 18S. The results from ovarian tissue from 3 separate brook trout females for each group are expressed as mean -ΔΔ*C*t ± SE (n = 3) with respect to the control, which has been set at 0. Statistically significant (p < 0.05) differences with respect to the control group are indicated by an asterisk (*).

In a previous study, we reported changes in ovarian function in trout that had been administered with LPS to simulate a bacterial infection [10]. Specifically, we hypothesized that the induction of follicular apoptosis in LPS-injected females and the stimulation of follicle contraction by LPS-stimulated macrophage conditioned medium could have been caused by the increased local production of pro-apoptotic cytokines, such as TNFα. Therefore, in order to determine if TNFα could mediate the observed effects of LPS in the trout ovary, in the present study we investigated the direct biological effects of rtTNFα on trout preovulatory follicles. Importantly, we first demonstrate here that LPS administration *in vivo *causes a significant induction (1.99-fold; p < 0.05) of the ovarian expression of TNFα in brook trout (Fig. 1), the species used in our previous study (8). We have also confirmed the stimulation of ovarian TNFα expression by LPS administration in rainbow trout (results not shown), which suggest that this effect may not be species-specific.

### Effects of rtTNFα on ovarian apoptosis and viability

**Figure 2:**
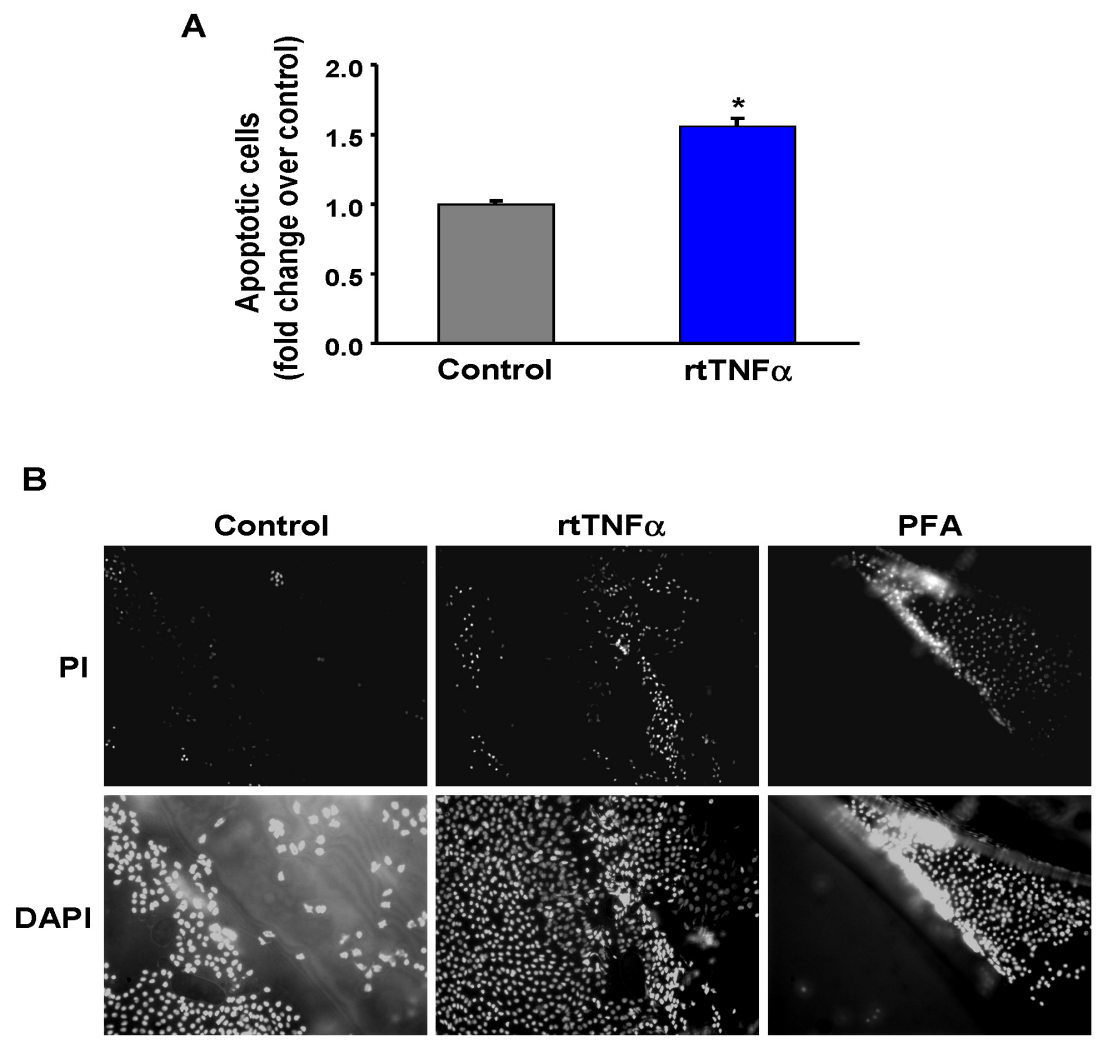
**Effects of rtTNFα treatment *in vitro *on apoptosis and cell viability in brown trout preovulatory follicles**. A. To determine the incidence of apoptosis, granulosa cells isolated from trout preovulatory follicles incubated in the absence or presence of rtTNFα (100 ng/ml) were stained with propidium iodide (PI) and analyzed by flow cytometry (FACS analysis). Each bar represents the mean ± SE of six independent experiments, each performed with ovarian tissue from a different female (n = 6). The results are expressed with respect to the control group which has been set at 1. Statistically significant (p < 0.05) differences with respect to the control group are indicated by an asterisk (*). B. Granulosa cell viability was assessed by incubating brown trout preovulatory follicles in the absence or presence of 100 ng/ml rtTNFα, or 1% paraformaldehyde (PFA) as a positive control, for 18 h at 15°C, separating the granulosa layers and staining them with PI. Subsequently, sheets of granulosa layers were mounted onto glass slides, counterstained with DAPI and visualized under a fluorescent microscope.

We investigated whether rtTNFα can induce apoptosis and/or cause a decrease in the viability of trout granulosa cells. Our results clearly show that the incidence of apoptotic granulosa cells significantly (p < 0.05) increased in trout ovarian follicles incubated with rtTNFα (Fig. 2A). Evaluation of granulosa cell viability by PI staining provided additional evidence that exposure of trout ovarian follicles to rtTNFα *in vitro *causes a marked decrease in granulosa cell viability (Fig. 2B). In contrast to the untreated controls in which almost no PI-positive cells were detected (Fig. 2B), treatment with rtTNFα increased the presence of PI-positive cells (Fig. 2B). Exposure of granulosa cells to 1% paraformaldehyde (PFA), used as a positive control, also caused a decrease in cell viability (Fig. 2B).

### Effects of rtTNFα on follicle contraction

**Figure 3:**
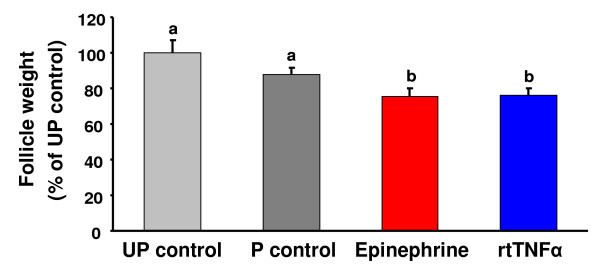
**Effects of rtTNFα treatment *in vitro *on follicle contraction in brown trout preovulatory follicles**. Preovulatory brown trout ovarian follicles were incubated for 16 h at 15°C in the presence or absence of the test compounds epinephrine and rtTNFα. Each bar represents the mean ± SE from three independent experiments, each performed with preovulatory follicles from a different female and assayed in triplicate. The results are expressed as percent change with respect to the unpunctured control group (UP control) which has been set at 100%. Punctured (P) trout preovulatory ovarian follicles were incubated in the absence or presence of epinephrine (10 μM) or rtTNFα (50 ng/ml). Statistically significant (p < 0.05) differences among groups are indicated by different letters.

Follicle contraction was significantly (p < 0.05) stimulated in isolated trout preovulatory follicles incubated in the presence of rtTNFα (Fig. 3), as evidenced by the decrease in follicle weight when compared with untreated unpunctured controls (UP controls) and punctured controls (P controls). Epinephrine (10 μM), used as a positive control, also significantly (p < 0.05) stimulated follicle contraction. There was approximately a 12% spontaneous contraction in punctured control follicles, as shown in previous studies [15,16].

### Effects of rtTNFα on steroid production

**Figure 4:**
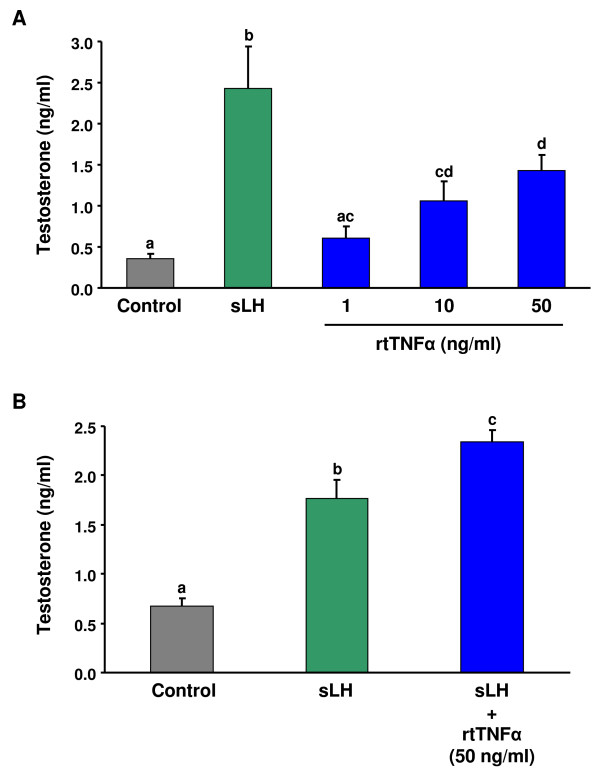
**Steroid production of brown trout preovulatory follicles treated with rtTNFα *in vitro***. Preovulatory trout follicles were incubated in the absence or presence of coho salmon LH (sLH; 25 ng/ml), different concentrations of rtTNFα (1, 10 and 50 ng/ml) (A) or sLH in the absence or presence of 50 ng/ml of rtTNFα (B) for 24 h at 15°C. Testosterone levels were measured in the medium at the end of the incubation period. Each bar represents the mean ± SE of two independent experiments, each with ovarian tissue from a separate female and assayed in triplicate. Statistically significant (p < 0.05) differences among groups are indicated by different letters.

Incubation with rtTNFα stimulated the *in vitro *production of T in a concentration-dependent manner (significantly at 10 and 50 ng/ml; p < 0.05) (Fig. 4A) by intact trout follicles. As expected, sLH (25 ng/ml) significantly (p < 0.05) stimulated the production of T. In the same manner, rtTNFα caused a significant (p < 0.05) potentiation of the effects of sLH on T production (Fig. 4B).

### Effects of rtTNFα on ovarian gene expression

In order to evaluate the effects of rtTNFα treatment on gene expression in the trout ovary, we used a salmonid fish cDNA microarray platform (SFA2.0) enriched with immune-related genes that has been previously validated for studies involving response to stress and toxicity [19,20] as well as the immune response in trout [10,23-25].

**Figure 5:**
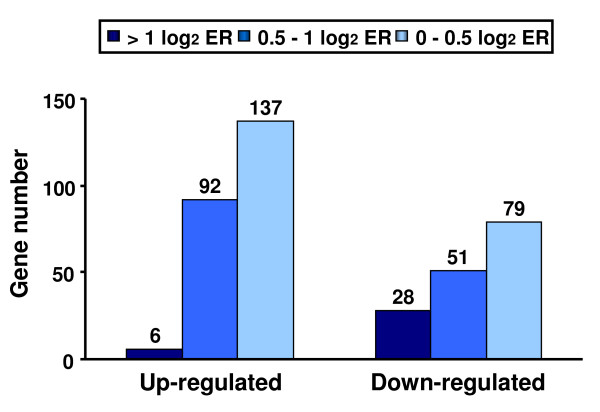
**Gene response to *in vitro *rtTNFα treatment of brown trout preovulatory follicles**. Total number of up- and down-regulated genes (> 0 log_2 _expression ratio, ER; p < 0.01) expressed in response to rtTNFα (100 ng/ml) treatment as assessed by microarray analysis using the SFA2.0 platform.

**Figure 6:**
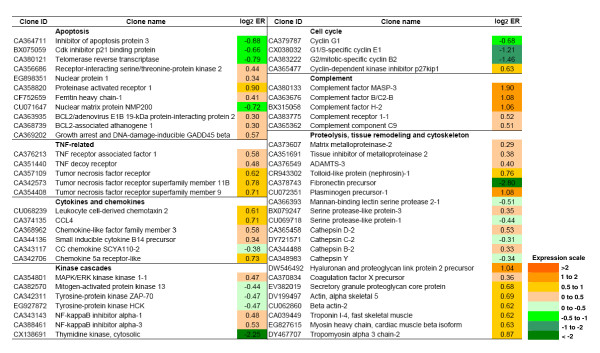
**Genes differentially regulated by rtTNFα in brown trout preovulatory follicles**. Brown trout preovulatory follicles incubated in the absence or presence of rtTNFα (100 ng/ml) were used for gene expression analysis using the SFA2.0 microarray. Data shown represent mean log_2 _ER (expression ratio). Significantly up- and down-regulated genes (p < 0.01, Student's t-test, 12 spot replicates per gene) are highlighted with color scale. See Additional File 1, Figure S1 for complete microarray data.

Overall, a larger number of genes were up-regulated (n = 235) rather than down-regulated (n = 158) in trout ovarian follicles in response to rtTNFα treatment (Fig. 5 and Additional File 1, Figure S1). However, applying a cut-off value of > 1 log_2 _ER (expression ratio) over the differentially expressed genes (DEGs) (p < 0.01) resulted in a considerably higher number of genes being down-regulated (n = 28) as opposed to those up-regulated (n = 6) (Fig. 5). Furthermore, the highest ERs in individual genes were observed among the down-regulated genes (Additional File 1, Figure S1). Fig. 6 shows examples of DEGs with known functions based on gene ontology.

**Figure 7:**
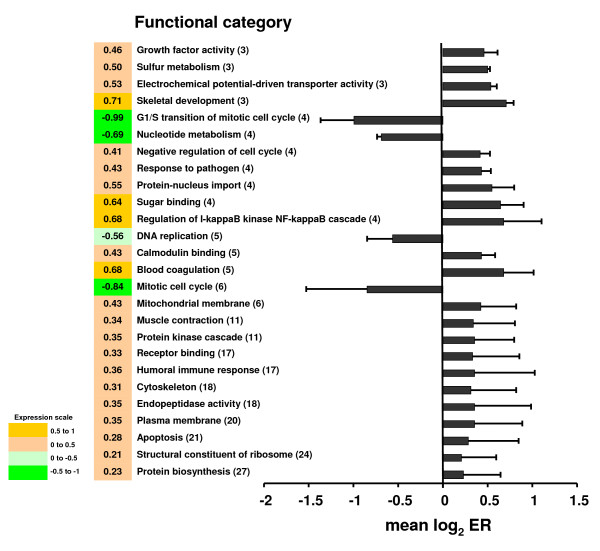
**Gene Ontology analysis of genes expressed in response to rtTNFα treatment in brown trout preovulatory follicles**. Differentially expressed genes were grouped by Gene Ontology functional categories and mean log_2 _ER (expression ratio) was analyzed by Student's t-test (p < 0.05). The number of regulated genes for each category is shown in parenthesis. Data is shown as mean log_2 _ER ± SE. The expression value is coded with color scale. See Additional File 2, Figure S2A and B for the complete microarray data.

The DEGs that were identified in response to rtTNFα treatment in the trout ovary were analyzed by interrogating the functional classes of Gene Ontology (GO) [21] and compared by the mean log_2 _ER of ranked genes (Student's t-test, p < 0.05) (Fig. 7 and Additional File 2, Figure S2A and B). The categories G1/S transition of mitotic cell cycle, nucleotide metabolism, DNA replication and mitotic cell cycle were reduced by rtTNFα, whereas protein biosynthesis and structural constituent of ribosome were enhanced. Also, categories related to protein kinase and I-kappaB kinase NF-kappaB cascades and apoptosis were up-regulated. Furthermore, categories involved in tissue remodeling, cytoskeleton and muscle contraction were also increased.

Microarray analyses showed that several genes involved in apoptosis underwent changes in response to rtTNFα *in vitro *administration (Fig. 6), in support of the observed increase in apoptosis in rtTNFα-treated ovarian follicles (Fig. 2). Receptor-interacting serine/threonine-protein kinase 2 (cell death protein RIP) and growth arrest and DNA-damage inducible GADD45 beta, involved in TNFα-induced apoptosis, were significantly up-regulated. TNF-dependent genes increased their expression, including several receptors (TNF receptor superfamily members). Also, inhibitor of apoptosis protein 3 and cdk inhibitor p21 binding protein were significantly down-regulated after rtTNFα treatment. In addition, the expression of telomerase reverse transcriptase and nuclear matrix protein NMP200 were also reduced in the trout ovary. In mammals, loss of expression of these two genes results in apoptosis and decreased cell survival after DNA damage [26,27]. Interestingly, genes involved in the cell cycle (such as cyclins) were reduced, in accordance with the GO categories regarding negative regulation of cell cycle and mitotic cell cycle.

Importantly, rtTNFα affected a number of genes involved in proteolysis, tissue remodeling and cytoskeleton (Fig. 6). This set of genes includes matrix metalloproteinase-2 (MMP-2 or gelatinase A), tissue inhibitor of metalloproteinase 2 (TIMP-2), plasminogen precursor-1, ADAMTS-3 (a disintegrin and metalloproteinase with thrombospondin-like motifs 3), serine proteases (serine protease-like protein-1 and 3), complement components (complement factor H-2, complement factor MASP-3, complement factor B/C2-B, complement component C9 and complement receptor 1-1), cathepsins (cathepsin D-2, C-2, B-2 and Y), cytoskeletal proteins and sarcomeric proteins involved in contractile function (actins, myosins and troponins).

Treatment with rtTNFα also caused an increase in the expression of immune-related genes in the trout ovary (Fig. 6 and Additional File 1, Figure S1). Several cytokines and chemokines, such as the small inducible cytokine B14 precursor (CXCL14), CCL4, leukocyte cell-derived chemotaxin 2 or chemokine-like factor family member 3 were induced. Furthermore, rtTNFα increased the expression of genes involved in communication and signaling, such as several MAP kinases and NF-kappaB inhibitor alpha transcripts (1 and 3). Also, genes coding for immune-related receptors showed changes in expression, such as chemokine 5a receptor-like, C type lectin receptor B and, as indicated above, several TNF receptor superfamily members.

In the present study, we selected eight differentially expressed genes representing varying levels of expression to verify the microarray results by qPCR (Table 2) using the same experimental treatments. Results obtained by qPCR validated the data obtained from microarray analysis. Genes such as small inducible cytokine B14 precursor, CCL4 and triosephosphate isomerase were highly regulated in the qPCR analysis.

**Table 2:** qPCR validation of microarray results for selected genes

Clone ID	Clone name	Regulation	Microarray	qPCR
CA344136	Small inducible cytokine B14 precursor	Up-regulated	0.33	1.33
CU068239	Leukocyte cell-derived chemotaxin 2	Up-regulated	0.61	0.41
CA374135	CCL4	Up-regulated	0.70	2.36
CA351440	TNF decoy receptor	Up-regulated	0.49	0.38
DW550559	Coronin-1C	Down-regulated	-1.18	-0.24
CU067298	Triosephosphate isomerase	Down-regulated	-1.28	-2.47
CU069718	Serine protease-like protein-1	Down-regulated	-0.43	-0.68
CA364711	Inhibitor of apoptosis protein 3	Down-regulated	-0.88	-1.24

Data from real-time PCR (qPCR) is shown as -ΔΔ*C*t (n = 3) and data from microarray as mean log_2 _ER (expression ratio).

Also, we identified by qPCR (Table 3) the expression of genes that have been postulated to be involved in proteolytic and tissue remodeling processes during ovulation in trout (i.e. kallikrein, KT-14; trout ovulatory protein-2, TOP-2; and novel osteopontin-like protein, NOP) [28-30]. The expression of this set of genes was significantly (p < 0.05) increased over control in response to rtTNFα treatment (100 ng/ml). Furthermore, we analyzed two interesting genes that are also expressed during the ovulatory process, such as macrophage migration inhibitory factor (MIF), a cytokine stimulated in response to human chorionic gonadotropin (hCG) or LH in follicular fluid [31], and TNFα trout decoy receptor (TDcR), a soluble receptor detected in the ovary during and after natural ovulation [32] (Table 3). The expression of both genes was significantly (p < 0.05) increased by rtTNFα.

**Table 3:** In vitro effects of rtTNFα (100 ng/ml) on candidate gene expression in brown trout preovulatory follicles

GenBank accession	Gene name	qPCR
AF005026	Kallikrein (KT-14)	1.01 *
AF223388	Novel osteopontin-like protein (NOP)	0.85 *
U67854	Trout ovulatory protein-2 (TOP-2)	0.60 *
AF156738	Trout decoy receptor (TDcR)	1.14 *
CA380218	Macrophage migration inhibitory factor (MIF)	1.17 *

The relative expression was determined by real-time PCR (qPCR) and normalized to the abundance of 18S. The results from follicles from 3 separate females are expressed as mean -ΔΔ*C*t (n = 3) with respect to the control, which has been set at 0 for each particular gene. Statistically significant (p < 0.05) differences with respect to the control group are indicated by an asterisk (*). Genes are: kallikrein (KT-14), NOP (novel osteopontin-like protein), TOP-2 (trout ovulatory protein-2), TDcR (trout decoy receptor), MIF (macrophage migration inhibitory factor).

## Discussion

In mammals, it has been hypothesized that TNFα may be a component of the ovulatory mechanism [33,34]. In fact, TNFα is produced in the ovary [35,36] and its role in steroidogenesis, proliferation and follicular apoptosis has been studied in several species [37,38]. Furthermore, ovulation is blocked after injection of TNFα antibodies into the follicular antrum [8]. All these observations suggest that TNFα plays multiple physiological roles in ovarian function in mammals. In order to study the possible involvement of TNFα in ovarian function in fish, we have investigated for the first time the effects of rtTNFα on ovarian function in a teleost fish, the brown trout (*Salmo trutta*). In particular, we examined the *in vitro *effects of rtTNFα treatment on ovarian apoptosis and viability, follicle contraction, steroid production and gene expression.

One of the most significant results obtained in the present study is the stimulation of trout granulosa cell apoptosis by rtTNFα, as reported in mammals [8], and the modulatory effects of this cytokine on the expression of apoptosis-related genes in trout preovulatory follicles. Interestingly, the pro-apoptotic effects of rtTNFα at the cellular and molecular levels parallel those reported in the ovary of LPS-treated female trout [10]. In fact, the expression of two anti-apoptotic genes (i.e. cdk inhibitor p21 binding protein and telomerase reverse transcriptase) in the preovulatory ovary was similarly down-regulated by *in vivo *administration of LPS and by *in vitro *treatment with rtTNFα. However, rtTNFα treatment also increased the expression of BCL-2 family members such as BCL2/adenovirus E1B 19-kDa protein-interacting protein 2 and BCL2-associated athanogene 1 (BAG1). BCL-2 interacting protein BAG-1 enhances the anti-apoptotic effects of BCL-2 [39] and expression of BCL-2 in mouse oocytes during fetal development suppresses apoptotic cell death [40]. Furthermore, the expression of ferritin heavy chain-1 was also increased by rtTNFα and its up-regulation is known to inhibit TNFα-induced apoptosis [41]. These results could suggest the existence of a balance between multiple pro-apoptotic (such as cell death protein RIP, growth arrest and DNA-damage inducible GADD45 beta or nuclear protein 1) and anti-apoptotic genes regulated by TNFα around the time of ovulation that could be important in order to exert a tight, and perhaps localized, control of TNFα-induced apoptosis in the trout ovary.

In addition to the induction of apoptosis, treatment of trout preovulatory follicles with rtTNFα caused important changes in the expression of genes involved in proteolysis and remodeling of the ECM. In mammals, alterations in the ECM of the follicle wall are an essential part of ovulation. For the oocyte to be released, extensive breakdown of the collagenous tissue of the theca externa and of the granulosa cell basement membrane, as well as dissociation of granulosa cell layers, are required [42]. In fish, there is also evidence for ECM remodeling during ovulation since it has been reported that MMPs with collagenase activity increase in the follicle wall at the time of ovulation in trout [43,44]. Furthermore, up-regulation of other candidate genes possibly involved in ovarian proteolysis such as kallikrein (KT-14), a serine protease postulated to regulate oocyte expulsion in trout [30], TOP-2, a serine protease inhibitor [29,45], as well as serine protease 23 and ADAM22, two genes identified by microarray analysis [46], occurs during natural ovulation in trout. In medaka (*Oryzias latipes*), several MMPs and TIMP-2 have been shown to play a crucial role in ECM remodeling, degradation of the follicle wall and, subsequently, ovulation, as in mammals [47]. Specifically, MMP-2 (gelatinase A) and membrane-type 2 MMP facilitate follicle rupture during ovulation by degrading type IV and type I collagen, respectively, and TIMP-2 regulates the production and activity of MMP-2 in medaka preovulatory follicles [47]. However, there is no evidence to date regarding the nature of the regulatory factors that control the production and activity of proteins involved in follicle rupture and ovulation in fish. In the present study we show that rtTNFα induces the expression of proteolytic enzymes in the trout ovary that belong to the three classes of proteinases known to be involved in ECM remodeling during ovulation in mammals: MMPs (e.g. MMP-2), plasmin/plasminogen activator system (e.g. plasminogen precursor-1) and ADAMTS (e.g. ADAMTS-3). In addition to these proteinases, rtTNFα induced the expression of TOP-2, TIMP-2, kallikrein and other serine proteases. Furthermore, we observed an increase in the expression of several cathepsins (D-2, C-2, B-2 and Y) in agreement with recent information suggesting an important role for cathepsins during ovarian follicle growth and maturation in fish [48]. Therefore, we have evidence supporting the hypothesis that rtTNFα may be an important factor regulating the degradation of the follicle wall, as a prerequisite for oocyte release, in the trout ovary.

Here we also provide direct evidence for a stimulatory role of rtTNFα on follicle contraction, a process required for oocyte expulsion during ovulation. Increased follicle contraction is most likely related to the observed up-regulation of the expression of cytoskeletal and sarcomeric proteins involved in contractile function. In a previous study we suggested that the stimulation of trout follicle contraction by conditioned medium from LPS-stimulated trout macrophages was due to the presence of inflammatory cytokines in the medium [10]. We have recently confirmed the ability of LPS-stimulated trout macrophages to secrete native TNFα protein [13] and, consequently, we can reasonably speculate that TNFα was at least one of the cytokines present in conditioned medium responsible for the stimulation of follicle contraction. In addition to TNFα, other factors such as chatecholamines and prostaglandins stimulate follicle contraction in trout [15,16]. Future studies in our laboratory are devoted to investigate a possible link between TNFα and prostaglandins in the stimulation of follicle contraction in trout preovulatory follicles.

The stimulation of testosterone production by rtTNFα in trout preovulatory follicles is interesting in relation to the known potentiatory role of androgens in the ovulatory process in mammals [49]. Although the precise mechanism by which androgens may induce ovulation in mammals is not well characterized, there is evidence for the increased expression of proteolytic enzymes such as kallikrein [30], suggesting that androgens could be involved in follicle rupture. In female salmonid fish (salmon and trout), testosterone plasma levels are known to increase significantly prior to ovulation [30,50] but there is no functional evidence for a role of testosterone in ovulation in fish. Our results, supported by the fact that ovarian follicles from LPS-treated trout showed increased sLH-stimulated testosterone production [10], suggest that TNFα could be responsible, at least in part, for the high ovarian testosterone output in preovulatory trout. It is tempting to speculate that TNFα-induced testosterone could play a role in the ovulatory process in fish.

Based on our observations on the stimulatory effects of rtTNFα on trout granulosa cell apoptosis and on the expression of genes involved in proteolysis of the trout follicle wall, we suggest that, as described in mammals (reviewed in [42]), TNFα in trout could facilitate ovulation by serving the dual role of promoting collagen degradation in the follicle wall and further disrupting it by deleting follicular cells by apoptosis. This role of TNFα, coupled with its ability to directly stimulate follicle contraction in trout preovulatory follicles, strongly suggests that TNFα may have pro-ovulatory actions in fish. In support of the proposed role for TNFα in the ovulatory process in fish, inhibition of the activity of TNFα-converting enzyme (TACE/ADAM17), which inhibits TNFα secretion by trout macrophages [13], blocks ovulation in isolated medaka follicles [47]. Furthermore, we have recently shown that LPS administration *in vivo *may result in an advancement of the time of ovulation in trout [51]. Having demonstrated that LPS administration *in vivo *induces the expression of TNFα in the trout ovary (this study), we can speculate that ovarian production of TNFα may trigger the ovulatory process. At least in trout, the expression of ligands and receptors of the TNF family, most notably TNFα itself [52] and TDcR [32], which may serve to bind TNFα and control its activity, increase at the time of ovulation.

## Conclusions

This is the first report in fish describing the possible role of TNFα in trout ovarian follicles during the preovulatory period. rtTNFα induced apoptosis in trout granulosa cells and altered the expression of genes known to be involved in the normal ovulatory process in trout, including genes involved in inflammation, proteolysis and tissue remodeling. Furthermore, rtTNFα stimulated the contraction of preovulatory trout follicles as well as the production of testosterone. These results suggest that TNFα, as in mammals, may be an important factor in the control of ovulation in fish by participating in the biomechanics of follicle weakening and rupture as well as in oocyte expulsion during ovulation. Furthermore, the results of the present study support the idea that bacterial pathogens, through the induction of pro-inflammatory cytokines such as TNFα, could have an impact on ovarian function, possibly resulting in an advancement of ovulation in fish, with important consequences for broodstock and egg quality in farmed fish.

## Competing interests

The authors declare that they have no competing interests.

## Authors' contributions

DC performed gene expression analyses, testosterone measurement, follicle contraction determinations and the writing of the manuscript. EB participated in the generation of the apoptosis and cell viability data. NR carried out the generation of recombinant TNFα. SM participated in the design of the study. AK participated in the microarray analysis. FWG participated in the design of the study and in the execution of the LPS experiment. JB participated in the design of the study, in execution of the experiments and in the writing of the manuscript. JVP coordinated and participated in the design of the study as well as in the execution of the experiments and drafted the manuscript. All authors read and approved the final manuscript.

## Supplementary Material

Additional file 1 Supplemental figure 1: Complete list of differentially expressed genes. Click here for file

Additional file 2 Supplemental figure 2A and B: Complete Gene Ontology analysis. Click here for file
