# Supplementary material for: Cellular and molecular evidence for a role of tumor necrosis factor alpha in the ovulatory mechanism of trout
Source: Reprod Biol Endocrinol. 2010 Apr 12;8:34. doi: 10.1186/1477-7827-8-34 (PMC2873445; doi:10.1186/1477-7827-8-34)
Supplement: Additional file 1 — Supplemental figure 1: Complete list of differentially expressed genes. [file 1477-7827-8-34-S1.PDF]

Clone ID	Clone name	log2 ER	p-value
CA380133	Complement factor MASP-3	1.90	0.00349
CA363676	Complement factor B/C2-B	1.08	0.00000
CU072351	Plasminogen precursor-1	1.08	0.00001
BX315058	Complement factor H-2	1.06	0.00108
CB511296	Unknown-247	1.05	0.00000
DW546492	Hyaluronan and proteoglycan link protein 2 precursor	1.04	0.00001
BX885863	C type lectin receptor B	0.97	0.00000
EG844993	Unknown-140	0.95	0.00059
DW538511	Hypothetical-fish 22	0.92	0.00027
EL554658	Thioredoxin-dependent peroxide reductase-1	0.90	0.00496
CA358820	Proteinase activated receptor 1	0.90	0.00173
BX078840	T-cell receptor alpha chain V region HPB-MLT precursor (Fragment)	0.89	0.00002
CA353313	Cytochrome B-245 heavy chain-1	0.87	0.00665
DY467707	Tropomyosin alpha 3 chain-2	0.87	0.00123
CX016176	Cytochrome c oxidase subunit I-1	0.84	0.00008
CA043257	MHC class 1b antigen	0.81	0.00000
CR363185	Ribosomal protein L6-1	0.80	0.00000
CA362787	Glomulin	0.78	0.00186
CA342573	Tumor necrosis factor receptor superfamily member 11B	0.78	0.00001
CA385359	Serum amyloid P-component-2	0.78	0.00125
CA368198	GDP-mannose 4,6 dehydratase	0.76	0.00186
BX859345	Unknown-157	0.76	0.00000
CR943302	Tolloid-like protein (nephrosin)-1	0.76	0.00117
CA364370	Tumor necrosis factor receptor superfamily member 5 precursor	0.75	0.00401
CX245400	Ependymin related protein-1	0.75	0.00013
CU070060	Unknown-191	0.75	0.00360
CA382877	Fibronectin receptor beta	0.74	0.00000
CA372642	Selectin L-like	0.74	0.00001
CA342706	Chemokine 5a receptor-like	0.73	0.00001
DW567981	26S protease regulatory subunit 6B-1	0.73	0.00812
CA374135	CCL4	0.71	0.00010
CA354408	Tumor necrosis factor receptor superfamily member 9	0.71	0.00011
EG780167	Unknown-199	0.71	0.00023
DV199497	Actin, alpha skeletal 5	0.69	0.00004
EV382019	Secretory granule proteoglycan core protein	0.68	0.00001
CA341808	Tax1 binding protein 1	0.67	0.00363
BX309855	28S ribosomal protein S16, mitochondrial precursor	0.67	0.00030
CR362431	Survival of motor neuron-related splicing factor 30	0.67	0.00316
CU070488	39S ribosomal protein L45, mitochondrial precursor	0.66	0.00484
CA359060	MHC class I antigen	0.66	0.00002
CU072414	Hypothetical-fish 33	0.65	0.00000
CA375694	Phosphotyrosine independent ligand for the Lck SH2 domain p62	0.65	0.00001
CA378035	Transport-associated protein	0.65	0.00028
BX299113	Unknown-149	0.65	0.00005
DY707573	Unknown-109	0.64	0.00000
BX299891	Hypothetical-fish 9	0.63	0.00051
EG827615	Myosin heavy chain, cardiac muscle beta isoform	0.63	0.00001
CA365477	Cyclin-dependent kinase inhibitor p27kip1	0.63	0.00220
CA370333	Melanoma derived growth regulatory protein	0.62	0.00005
CA039449	Troponin I-4, fast skeletal muscle	0.62	0.00636
EG794922	Hypothetical-fish 27	0.62	0.00013
CU062860	Beta actin-2	0.62	0.00320
CA357109	Tumour necrosis factor receptor	0.62	0.00000
CU068239	Leukocyte cell-derived chemotaxin 2	0.61	0.00000
CA373890	Heat shock protein HSP 90-beta-1	0.61	0.00000

DV106774	Guanine nucleotide-binding protein beta subunit 2-like 1	0.60	0.00073
CA062642	Hypothetical-fish 24	0.60	0.00095
BX869335	Fructose-1,6-bisphosphatase isozyme 2	0.59	0.00001
EG828714	Actin, alpha skeletal 4	0.59	0.00010
BX890112	Cytochrome P450 2F1	0.59	0.00215
CB493870	Unknown-185	0.59	0.00003
CX144401	Proteasome subunit alpha type 6	0.59	0.00000
CX145944	Unknown-158	0.58	0.00389
CX723644	Hypothetical-fish 7	0.58	0.00000
CA376213	TNF receptor associated factor 1	0.58	0.00012
CA385071	Sodium/bile acid cotransporter	0.58	0.00142
CA368962	Chemokine-like factor family member 3	0.58	0.00055
CX141838	Lysozyme g-3	0.58	0.00001
CA376574	Peptidyl-prolyl cis-trans isomerase 1	0.58	0.00065
CA350333	Ras activator RasGRP	0.58	0.00110
CA355055	Reversion-inducing cysteine-rich protein with Kazal motifs	0.58	0.00039
CA384463	B-cell translocation gene 1-1	0.57	0.00265
CT563975	ADP,ATP carrier protein T2	0.57	0.00165
EL554685	Basic leucine-zipper protein BZAP45-1	0.57	0.00001
CA369202	Growth arrest and DNA-damage-inducible GADD45 beta	0.57	0.00000
CR375542	Hypothetical-fish 10	0.55	0.00289
CX151839	TGF beta-inducible nuclear protein 1	0.55	0.00207
CU063987	Heterogeneous nuclear ribonucleoprotein A0	0.55	0.00008
CX147525	Prostaglandin D synthase homolog	0.55	0.00089
CA344707	Cyclophilin-1	0.54	0.00084
CX351269	Unknown-196	0.54	0.00145
CU063954	Trans-2-enoyl-CoA reductase	0.54	0.00096
CA385699	Aflatoxin B1 aldehyde reductase member 2	0.53	0.00110
DN166158	Myosin heavy chain, skeletal, adult 1-2	0.53	0.00140
CR364270	All-trans-13,14-dihydroretinol saturase	0.53	0.00006
CA375106	Protein-tyrosine sulfotransferase 1	0.53	0.00292
CA365458	Cathepsin D-2	0.53	0.00005
CA388461	NF-kappaB inhibitor alpha-3	0.53	0.00005
CA371855	PDZ and LIM domain protein 1	0.52	0.00003
CA383775	Complement receptor 1-1	0.52	0.00035
BX084971	Myosin light chain 2-1	0.52	0.00127
ES325544	Creatine kinase, M-3	0.51	0.00576
CA365362	Complement component C9	0.51	0.00888
BX876267	Hypothetical-fish 18	0.51	0.00001
BX318049	Unknown-202	0.51	0.00052
DN165723	Elongation factor 1-alpha 2	0.50	0.00422
CB503250	Ribosomal protein L6-2	0.50	0.00003
EG895745	60S ribosomal protein L18	0.50	0.00001
CA361754	Glutathione S-transferase theta 1	0.49	0.00088
CA349338	Unknown-275	0.49	0.00407
CX247997	Dermatan-4-sulfotransferase-1-2	0.49	0.00014
CX144614	Transferrin	0.48	0.00032
CA351440	TNF decoy receptor	0.48	0.00863
EV384694	Dermatan-4-sulfotransferase-1-1	0.48	0.00001
BX307069	Tumor differentially expressed protein 1	0.48	0.00191
CA343143	NF-kappaB inhibitor alpha-1	0.48	0.00553
BX078446	Hypothetical-fish 23	0.47	0.00015
CA354801	MAPK/ERK kinase kinase 1-1	0.47	0.00011
CX724354	60S ribosomal protein L7a-2	0.47	0.00003
BX307506	NADH-ubiquinone oxidoreductase 15 kDa subunit	0.47	0.00029
CA386037	B-cell receptor-associated protein 31	0.47	0.00162

CA361415	C-Jun protein	0.46	0.00042
CA374217	Alpha-globin 1-3	0.46	0.00043
BX307487	Unknown-168	0.46	0.00117
CA353365	Heat shock 70kD protein 9B-1	0.46	0.00024
CR375024	Eukaryotic translation elongation factor 1 alpha 1	0.45	0.00002
CA377504	Cold autoinflammatory syndrome 1 protein	0.45	0.00228
CA382657	T-complex protein 1, alpha subunit	0.45	0.00023
CA350788	Goodpasture antigen-binding protein	0.45	0.00005
CA387646	4F2 cell-surface antigen heavy chain	0.45	0.00216
CA359083	Unknown-83	0.45	0.00001
BX076335	Creatine kinase, M-2	0.45	0.00000
BX912124	Ribosomal protein S2	0.45	0.00000
CA344745	B-cell receptor CD22-1	0.44	0.00005
CA356686	Receptor-interacting serine/threonine-protein kinase 2	0.44	0.00008
CX724055	Prothymosin alpha	0.44	0.00436
BX082584	Complement factor H-1	0.44	0.00051
CA386271	Telomerase-binding protein p23-2	0.44	0.00010
CV428906	Cytochrome b-3	0.44	0.00029
CA379474	Glucose-6-phosphate isomerase-2	0.44	0.00000
CX247269	Unknown-198	0.44	0.00003
CA365039	CD63	0.43	0.00007
CA356429	NACHT-, LRR- and PYD-containing protein 2	0.43	0.00005
CA356239	Unknown-179	0.43	0.00098
CU064850	Galectin-3	0.43	0.00026
BX080469	Unknown-123	0.43	0.00063
BX876624	Hypothetical-fish 4	0.42	0.00015
CK876338	Gamma crystallin B-1	0.42	0.00033
CA364434	CYR61 protein	0.42	0.00169
CF752659	Ferritin heavy chain-1	0.41	0.00048
CA363852	Double stranded RNA adenosine deaminase RED2	0.41	0.00000
DY738845	Heat shock cognate 70 kDa	0.41	0.00010
BX879808	Myosin heavy chain, skeletal, adult 1-1	0.41	0.00002
CA046645	Unknown-150	0.41	0.00030
CA359442	Liver-expressed antimicrobial peptide 2A	0.41	0.00286
CA365505	Retinoblastoma-like protein 1	0.40	0.00272
CA362302	Guanylate-binding protein	0.40	0.00029
CA376549	ADAMTS-3	0.40	0.00027
BX876605	D-3-phosphoglycerate dehydrogenase	0.40	0.00256
CX262645	Unknown-121	0.40	0.00030
CA368622	T-complex protein 1, subunit 5	0.40	0.00469
CX151275	Estrogen-responsive B box protein	0.40	0.00001
EV376689	Nuclease sensitive element binding protein 1-1	0.40	0.00003
EG899519	Heat shock cognate 71 kDa protein	0.40	0.00027
CX149208	Annexin IV	0.40	0.00159
CA362691	N-myc downstream regulated protein-1	0.39	0.00047
EG902248	Unknown-232	0.39	0.00411
EG828714	Actin, alpha skeletal 3	0.39	0.00113
DV192348	Unknown-161	0.39	0.00002
CX254770	MIR-interacting saposin-like protein precursor	0.39	0.00054
CA351691	Tissue inhibitor of metalloproteinase 2	0.38	0.00243
CX351396	Full-length cDNA clone CS0DC006YH13 of Neuroblastoma of Homo sapiens	0.38	0.00008
EL561042	Heterogeneous nuclear ribonucleoprotein L	0.37	0.00077
EG902593	Zinc finger protein 228	0.37	0.00142
BX878953	Angiotensin I converting enzyme	0.37	0.00001
EG836496	Unknown-267	0.37	0.00490
CX028287	Ubiquitin-like protein SMT3A-2	0.37	0.00001

CA370834	Coagulation factor X precursor	0.36	0.00409
CA348284	CCAAT/enhancer binding protein beta	0.36	0.00053
CX137254	Hyperosmotic protein 21	0.36	0.00003
CF752415	Cytochrome c oxidase subunit I-2	0.35	0.00016
CA388407	DnaJ homolog subfamily A member 2	0.35	0.00052
CX042463	Receptor for activated C kinase	0.35	0.00000
CA347041	Cathepsin D-1	0.35	0.00027
CX145859	Troponin T-3, fast skeletal muscle	0.35	0.00177
CA359144	Aldehyde dehydrogenase 1A2	0.35	0.00326
BX079247	Serine protease-like protein-3	0.35	0.00003
BX079674	UDP-glucose 4-epimerase	0.35	0.00147
CX140273	Unknown-194	0.34	0.00677
EG923080	Chromosome-associated kinesin KIF4A	0.34	0.00254
EG898351	Nuclear protein 1	0.34	0.00016
CA384063	IkappaB kinase complex-associated protein-2	0.34	0.00489
BX316728	Ubiquitin	0.34	0.00391
DW472665	Myosin regulatory light chain MRCL2-2	0.34	0.00257
BX867772	Beta-galactosidase-related protein	0.34	0.00314
EG828714	Actin, alpha skeletal 2	0.34	0.00110
CU071907	Fumarate hydratase, mitochondrial precursor	0.34	0.00014
CB492144	Myristoylated alanine-rich protein kinase C substrate	0.34	0.00002
CA344136	Small inducible cytokine B14 precursor	0.34	0.00001
CA350956	Interferon regulatory factor 1-1	0.34	0.00024
DW473667	40S ribosomal protein S6	0.33	0.00138
CA358347	Transposase-31	0.33	0.00395
CA344488	Cathepsin B-2	0.33	0.00000
EG937246	40S ribosomal protein S3-1	0.33	0.00046
CF752992	Annexin 5	0.33	0.00006
CA341823	Superoxide dismutase [Cu-Zn] extracellular	0.32	0.00423
BX081480	Alanine-glyoxylate aminotransferase 1	0.32	0.00188
CA378853	C-terminal binding protein 1	0.32	0.00004
DW536420	Midkine precursor	0.32	0.00041
CX260733	Unknown-97	0.32	0.00000
CX261029	Unknown-176	0.31	0.00604
CA385248	Heat shock 70kD protein 9B-2	0.31	0.00552
CX153837	Eukaryotic translation initiation factor 3 subunit 6-2	0.31	0.00571
DY699171	Unknown-239	0.31	0.00216
CA363935	BCL2/adenovirus E1B 19-kDa protein-interacting protein 2	0.30	0.00394
CA368739	BCL2-associated athanogene 1	0.30	0.00571
CA385900	Nonhistone chromosomal protein HMG-17	0.30	0.00141
CX137536	Antifreeze protein LS-12	0.30	0.00116
BX079855	B-cell receptor-associated protein BAP37-2	0.30	0.00613
CA386586	Retinoic acid receptor RXR-beta	0.30	0.00211
CA379612	NF-kappaB inhibitor alpha-2	0.29	0.00280
CA365571	Lysyl oxidase homolog 2	0.29	0.00592
CA377887	Derlin-1	0.29	0.00036
AM041506	60S ribosomal protein L7	0.29	0.00814
CA373607	Matrix metalloproteinase-2	0.29	0.00001
CA385697	X-box binding protein 1	0.29	0.00101
BX077754	Creatine kinase, B chain	0.28	0.00011
DY726066	Ribosomal protein L13	0.28	0.00644
CX142615	60S ribosomal protein L10-1	0.28	0.00337
BX869823	40S ribosomal protein S11	0.28	0.00739
BX883167	Histone H33-3	0.27	0.00136
EG930585	Protein phosphatase 2C gamma isoform	0.27	0.00014
BX297527	Quinone oxidoreductase	0.27	0.00392

BX076465	Heat shock protein 75 kDa-2	0.26	0.00114
CA387992	Heparanase	0.26	0.00026
CA376378	Interferon-related regulator 1-2	0.26	0.00107
CA383094	Regulator of G-protein signaling 1-1	0.23	0.00060
CA356374	Unknown-145	0.22	0.00124
BX315722	NADH-ubiquinone oxidoreductase 19 kDa subunit	0.22	0.00842
CA368189	MAPK/ERK kinase kinase 6	0.22	0.00024
EB174216	60S ribosomal protein L32-2	0.22	0.00757
CX026066	Na/K ATPase alpha subunit-1	0.21	0.00573
CA384134	G1/S-specific cyclin D2	0.19	0.00735
BX874525	Unknown-201	0.17	0.00385
BX079548	Glyceraldehyde-3-phosphate dehydrogenase-1	0.15	0.00458
DR695415	60S ribosomal protein L36	-0.15	0.00130
BX074486	Heat shock protein HSP 90-beta-2	-0.15	0.00571
CA353121	GRB2-related adaptor protein 2	-0.16	0.00999
CX717338	Transposase-5	-0.18	0.00437
EV381335	40S ribosomal protein S14 (PRO2640)	-0.18	0.00743
DV200529	Fructose-bisphosphate aldolase A	-0.18	0.00065
CA362555	Transposase-40	-0.21	0.00081
CA355516	Transposase-26	-0.21	0.00733
CX254566	Unknown-76	-0.22	0.00905
CA382275	Egl nine homolog 2	-0.23	0.00004
EG875445	40S ribosomal protein S9-1	-0.23	0.00380
CU065234	Beta-globin	-0.23	0.00977
CX718505	Ribosomal protein L11	-0.25	0.00137
CA362149	Transposase-39	-0.26	0.00082
CA358884	Transposase -65	-0.27	0.00570
CA361474	Transposase-35	-0.27	0.00056
CA366800	Transposase-45	-0.27	0.00316
CA378361	Ubiquitin ligase SIAH1	-0.27	0.00004
CA351516	Reverse transcriptase-like-2	-0.27	0.00053
CA343327	Transposase -61	-0.27	0.00492
DW576495	Transposase-56	-0.27	0.00482
CA347342	Transposase-14	-0.28	0.00007
CA370086	Transposase-47	-0.28	0.00058
BX074146	Thymosin beta-4-2	-0.28	0.00188
CA356752	Transposase-27	-0.28	0.00003
CA359321	Phosphatidylinositol 3,4,5-trisphosphate-dependent Rac exchanger 1 protein	-0.28	0.00079
CA343612	ReO_6-3	-0.29	0.00109
CA356055	Transposase -63	-0.29	0.00037
CA341755	Transposase-9	-0.30	0.00034
CA359569	Voltage-dependent anion-selective channel protein 1	-0.30	0.00690
CX252245	Hypothetical-fish 34	-0.30	0.00074
CA347167	Transposase-13	-0.30	0.00436
CA348049	Low affinity immunoglobulin gamma Fc region receptor II-c	-0.31	0.00492
DY721571	Cathepsin C-2	-0.31	0.00837
EG938935	SAC2 suppressor of actin mutations 2-like	-0.31	0.00014
BX076826	Unknown-113	-0.33	0.00078
CA348983	Cathepsin Y	-0.34	0.00591
CX720733	Keratin, type I cytoskeletal 9	-0.34	0.00190
CA357749	Transposase-30	-0.35	0.00013
BX082905	Splicing factor 3B subunit 1	-0.35	0.00574
EV378274	Adenosine deaminase 3	-0.35	0.00032
CA368203	Transposase-55	-0.35	0.00892
CF752235	Ig mu heavy chain disease protein	-0.35	0.00438
EG847896	Transposase-6	-0.36	0.00052

CA346079	Transposase-48	-0.37	0.00019
CA343117	CC chemokine SCYA110-2	-0.38	0.00179
CA352734	Interferon regulatory factor 1-2	-0.38	0.00015
CX144768	60S ribosomal protein L5-1	-0.39	0.00154
CA356007	Transposase-53	-0.39	0.00003
CA366383	Unknown-78	-0.39	0.00000
CA350038	Transposase-18	-0.41	0.00104
CA344354	Unknown-273	-0.41	0.00010
EV378980	Alpha-2,8-sialyltransferase 8F	-0.42	0.00003
CR943429	MHC class II invariant chain-like protein 1	-0.42	0.00049
CA383564	Coatomer epsilon subunit 1	-0.42	0.00002
CB493099	Apolipoprotein E-2	-0.42	0.00023
CA350330	Unknown-276	-0.42	0.00001
BX076899	Nucleolar protein Nop56-2	-0.42	0.00032
CX153181	Nucleoside diphosphate kinase, mitochondrial precursor	-0.42	0.00006
CA352526	Transposase-22	-0.42	0.00002
CA342145	RNA binding motif protein 4	-0.42	0.00016
EL558025	Unknown-75	-0.43	0.00096
CA360844	Transposase-23	-0.43	0.00004
CA362806	Gamma-interferon inducible lysosomal thiol reductase	-0.43	0.00020
CA361817	Transposase-36	-0.43	0.00501
DY720828	Unknown-70	-0.43	0.00002
CU069718	Serine protease-like protein-1	-0.44	0.00111
CA346623	Selenium-binding protein 1	-0.44	0.00745
CA382570	Mitogen-activated protein kinase 13	-0.44	0.00000
CA342707	Unknown-272	-0.46	0.00050
CU064429	Mitochondrial ribosomal protein L4, isoform a	-0.46	0.00595
CA369915	Transposase-46	-0.46	0.00039
CX136217	ReO_6-2	-0.46	0.00018
CA342311	Tyrosine-protein kinase ZAP-70	-0.47	0.00805
EG927872	Tyrosine-protein kinase HCK	-0.47	0.00661
CA363737	Transposase-41	-0.47	0.00000
CA352430	Transposase-20	-0.48	0.00219
DY737056	Transposase-1	-0.48	0.00000
CA355265	Transposase-52	-0.49	0.00001
CX146291	Cation-transporting ATPase	-0.50	0.00014
CA366393	Mannan-binding lectin serine protease 2-1	-0.51	0.00020
CA346784	Hypothetical-fish 17	-0.51	0.00789
DW564653	Hpa repeat-1	-0.52	0.00013
DY708322	Glyceraldehyde-3-phosphate dehydrogenase-6	-0.52	0.00001
CA370329	Lysozyme C precursor	-0.52	0.00172
CU063628	Proteasome subunit alpha type 7-1	-0.53	0.00301
BX072809	Transcription elongation factor A protein 1	-0.54	0.00803
CU063237	Unknown-79	-0.55	0.00745
CA368533	Vitronectin	-0.56	0.00060
CA351948	Diacylglycerol kinase delta2	-0.56	0.00011
CA362032	Transposase-37	-0.57	0.00030
CA342656	Reverse transcriptase-like-1	-0.57	0.00012
CA363230	Hemopexin	-0.58	0.00059
CA370661	Barrier-to-autointegration factor	-0.60	0.00057
CX720153	40S ribosomal protein S20	-0.60	0.00000
CA370733	Microtubule-associated protein RP/EB	-0.62	0.00001
CA351460	Vitellogenin-1	-0.62	0.00099
CA367029	Rho guanine nucleotide exchange factor 5	-0.64	0.00262
CX354900	Transposase-59	-0.64	0.00000
BX075059	Cdk inhibitor p21 binding protein	-0.66	0.00001

CA385591	DnaJ homolog subfamily B member 11 precursor	-0.67	0.00091
CA351182	Glyoxylate reductase/hydroxypyruvate reductase	-0.68	0.00258
CA379787	Cyclin G1	-0.68	0.00007
CA384418	Ig heavy chain V region 5A	-0.69	0.00013
CA367667	FYVE finger-containing phosphoinositide kinase	-0.70	0.00132
CA346256	Transaldolase	-0.71	0.00008
BX077835	Laminin alpha-4 chain precursor	-0.71	0.00009
CX039326	Ubiquitin-like protein SMT3A-1	-0.71	0.00027
BX078994	COP9 signalosome complex subunit 6	-0.72	0.00025
CU071647	Nuclear matrix protein NMP200	-0.72	0.00233
BX857887	Red cell acid phosphatase 1	-0.73	0.00773
DY691980	Glyceraldehyde-3-phosphate dehydrogenase-4	-0.73	0.00198
CA366835	Serum albumin precursor	-0.74	0.00100
CA346925	Acidic leucine-rich nuclear phosphoprotein 32 E	-0.76	0.00002
CK883616	Calpactin I light chain	-0.77	0.00000
CA380121	Telomerase reverse transcriptase	-0.79	0.01000
CA368666	Brefeldin A-inhibited guanine nucleotide-exchange protein 1	-0.80	0.00264
CR370883	Hpa repeat-2	-0.80	0.00001
CA368411	Phosphatidylethanolamine N-methyltransferase, isoform 1	-0.80	0.00003
DY734542	Sp1 transcriptional activation factor	-0.81	0.00422
CX145328	Unknown-42	-0.81	0.00001
CA383652	ATP-binding cassette, sub-family F, member 1	-0.82	0.00246
CA343473	C3a anaphylatoxin chemotactic receptor	-0.83	0.00080
DV194438	Hypothetical-fish 44	-0.85	0.00009
CK883491	Unknown-240	-0.85	0.00002
CA364711	Inhibitor of apoptosis protein 3	-0.88	0.00369
BX307715	60 kDa heat shock protein-2	-0.88	0.00995
BX885774	Ribonucleoside-diphosphate reductase large subunit	-0.89	0.00006
CA355951	Alpha-taxilin	-0.89	0.00007
CA362042	Transposase-38	-0.94	0.00986
CA385421	Hypothetical-fish 45	-1.00	0.00196
CX260228	Hypothetical-fish 5	-1.02	0.00005
CB517450	Unknown-142	-1.02	0.00345
CA384452	Heat shock protein HSP 90-alpha	-1.03	0.00002
EG829337	Peroxiredoxin 2	-1.06	0.00095
EV378994	WD-repeat containing protein Ciao 1	-1.07	0.00177
CA371024	Hypothetical-fish 19	-1.08	0.00958
CA380135	Aldeyde oxidase	-1.10	0.00053
CA345105	Aldehyde dehydrogenase 7	-1.11	0.00540
CA345951	Hypothetical-fish 42	-1.14	0.00276
CA385882	Vesicle-associated membrane protein-associated protein B/C	-1.15	0.00334
DW550559	Coronin-1C	-1.18	0.00004
CX038032	G1/S-specific cyclin E1	-1.21	0.00000
CU067298	Triosephosphate isomerase	-1.28	0.00031
CX246241	Xaa-Pro dipeptidase	-1.30	0.00001
CB507951	Metallothionein-IL	-1.30	0.00001
BX856419	Unknown-96	-1.35	0.00271
CX035528	TATA box-binding protein	-1.37	0.00139
CA371435	Vasopressin-activated calcium-mobilizing receptor	-1.41	0.00023
CX248060	Similar to rRNA (Vangl2)	-1.42	0.00727
CA383222	G2/mitotic-specific cyclin B2	-1.46	0.00007
BX081739	Selenoprotein W	-1.47	0.00562
EG874928	Unknown-160	-1.57	0.00964
CB515179	TCF3 (E2A) fusion partner	-1.75	0.00003
CA381677	Beta-arrestin 2	-2.06	0.00462
CA364608	Hypothetical-fish 28	-2.11	0.00110

CX138691	Thymidine kinase, cytosolic	-2.25	0.00798
CA378743	Fibronectin precursor	-2.80	0.00000
