# Supplementary material for: Cellular and molecular evidence for a role of tumor necrosis factor alpha in the ovulatory mechanism of trout
Source: Reprod Biol Endocrinol. 2010 Apr 12;8:34. doi: 10.1186/1477-7827-8-34 (PMC2873445; doi:10.1186/1477-7827-8-34)
Supplement: Additional file 2 — Supplemental figure 2A and B: Complete Gene Ontology analysis. [file 1477-7827-8-34-S2.PDF]

A

Functional category	Genes	mean log2 ER	p-value	SE
Growth factor activity	3	0.46	0.03	0.15
Sulfur metabolism	3	0.50	0.00	0.03
Electrochemical potential-driven transporter activity	3	0.53	0.01	0.07
Skeletal development	3	0.71	0.00	0.08
G1 S transition of mitotic cell cycle	4	-0.99	0.01	0.38
Nucleotide metabolism	4	-0.69	0.05	0.42
Negative regulation of cell cycle	4	0.41	0.01	0.11
Response to pathogen	4	0.43	0.00	0.10
Protein-nucleus import	4	0.55	0.02	0.25
Sugar binding	4	0.64	0.02	0.26
Regulation of I-kappaB kinase NF-kappaB cascade	4	0.68	0.01	0.19
DNA replication	5	-0.56	0.01	0.28
Calmodulin binding	5	0.43	0.00	0.16
Blood coagulation	5	0.68	0.01	0.33
Mitotic cell cycle	6	-0.84	0.03	0.68
Mitochondrial membrane	6	0.43	0.05	0.39
Muscle contraction	11	0.34	0.04	0.47
Protein kinase cascade	11	0.35	0.02	0.44
Receptor binding	17	0.33	0.02	0.52
Humoral immune response	17	0.36	0.04	0.67
Cytoskeleton	18	0.31	0.02	0.51
Endopeptidase activity	18	0.35	0.03	0.63
Plasma membrane	20	0.35	0.01	0.54
Apoptosis	21	0.28	0.03	0.56
Structural constituent of ribosome	24	0.21	0.01	0.39
Protein biosynthesis	27	0.23	0.01	0.42

B

Clone name	log2 ER	mean log2 ER
**Growth factor activity (3)**		
Melanoma derived growth regulatory protein	0.62	0.46
Midkine precursor	0.32	
Glucose-6-phosphate isomerase-2	0.44	
**Sulfur metabolism (3)**		
Protein-tyrosine sulfotransferase 1	0.53	0.50
Dermatan-4-sulfotransferase-1-1	0.48	
Dermatan-4-sulfotransferase-1-2	0.49	
**Electrochemical potential-driven transporter activity (3)**		
Sodium/bile acid cotransporter	0.58	0.53
4F2 cell-surface antigen heavy chain	0.45	
ADP,ATP carrier protein T2	0.57	
**Skeletal development (3)**		
Tumor necrosis factor receptor superfamily member 11B	0.78	0.71
Chemokine 5a receptor-like	0.73	
Leukocyte cell-derived chemotaxin 2	0.61	
**G1/S transition of mitotic cell cycle (4)**		
Vasopressin-activated calcium-mobilizing receptor	-1.41	-0.99
Cyclin G1	-0.68	
Cdk inhibitor p21 binding protein	-0.66	
G1/S-specific cyclin E1	-1.21	
**Nucleotide metabolism (4)**		

Transaldolase	-0.71	-0.69
Adenosine deaminase 3	-0.35	
Triosephosphate isomerase	-1.28	
Nucleoside diphosphate kinase, mitochondrial precursor	-0.42	
**Negative regulation of cell cycle (4)**		
Interferon regulatory factor 1-1	0.34	0.41
Reversion-inducing cysteine-rich protein with Kazal motifs	0.58	
Retinoblastoma-like protein 1	0.40	
Fumarate hydratase, mitochondrial precursor	0.34	
**Response to pathogen (4)**		
NF-kappaB inhibitor alpha-1	0.48	0.43
NACHT-, LRR- and PYD-containing protein 2	0.43	
NF-kappaB inhibitor alpha-2	0.29	
NF-kappaB inhibitor alpha-3	0.53	
**Protein-nucleus import (4)**		
NF-kappaB inhibitor alpha-1	0.48	0.55
Proteinase activated receptor 1	0.90	
NF-kappaB inhibitor alpha-2	0.29	
NF-kappaB inhibitor alpha-3	0.53	
**Sugar binding (4)**		
B-cell receptor CD22-1	0.44	0.64
C type lectin receptor B	0.97	
Galectin-3	0.43	
Selectin L-like	0.74	
**Regulation of I-kappaB kinase NF-kappaB cascade (4)**		
Receptor-interacting serine/threonine-protein kinase 2	0.44	0.68
Proteinase activated receptor 1	0.90	
Tumor necrosis factor receptor superfamily member 5 precursor	0.75	
Phosphotyrosine independent ligand for the Lck SH2 domain p62	0.65	
**DNA replication (5)**		
Reverse transcriptase-like-1	-0.57	-0.56
ReO_6-3	-0.29	
Reverse transcriptase-like-2	-0.27	
Telomerase reverse transcriptase	-0.79	
Ribonucleoside-diphosphate reductase large subunit	-0.89	
**Calmodulin binding (5)**		
Regulator of G-protein signaling 1-1	0.23	0.43
Myristoylated alanine-rich protein kinase C substrate	0.34	
Myosin heavy chain, skeletal, adult 1-1	0.41	
Myosin heavy chain, cardiac muscle beta isoform	0.63	
Myosin heavy chain, skeletal, adult 1-2	0.53	
**Blood coagulation (5)**		
Proteinase activated receptor 1	0.90	0.68
Tumor necrosis factor receptor superfamily member 5 precursor	0.75	
Annexin 5	0.33	
Coagulation factor X precursor	0.36	
Plasminogen precursor-1	1.08	
**Mitotic cell cycle (6)**		
Vasopressin-activated calcium-mobilizing receptor	-1.41	-0.84
Cyclin G1	-0.68	
G2/mitotic-specific cyclin B2	-1.46	
DnaJ homolog subfamily A member 2	0.35	
Cdk inhibitor p21 binding protein	-0.66	
G1/S-specific cyclin E1	-1.21	
**Mitochondrial membrane (6)**		
Voltage-dependent anion-selective channel protein 1	-0.30	0.43

Cytochrome c oxidase subunit I-1	0.84	
ADP,ATP carrier protein T2	0.57	
Cytochrome c oxidase subunit I-2	0.35	
39S ribosomal protein L45, mitochondrial precursor	0.66	
Cytochrome b-3	0.44	
**Muscle contraction (11)**		
C3a anaphylatoxin chemotactic receptor	-0.83	0.34
Troponin T-3, fast skeletal muscle	0.35	
Fructose-bisphosphate aldolase A	-0.18	
Actin, alpha skeletal 2	0.34	
Actin, alpha skeletal 3	0.39	
Myosin heavy chain, skeletal, adult 1-1	0.41	
Tropomyosin alpha 3 chain-2	0.87	
Myosin heavy chain, cardiac muscle beta isoform	0.63	
Troponin I-4, fast skeletal muscle	0.62	
Actin, alpha skeletal 4	0.59	
Myosin heavy chain, skeletal, adult 1-2	0.53	
**Protein kinase cascade (11)**		
Tyrosine-protein kinase ZAP-70	-0.47	0.35
NF-kappaB inhibitor alpha-1	0.48	
Receptor-interacting serine/threonine-protein kinase 2	0.44	
Proteinase activated receptor 1	0.90	
Tumor necrosis factor receptor superfamily member 5 precursor	0.75	
MAPK/ERK kinase kinase 6	0.22	
Growth arrest and DNA-damage-inducible GADD45 beta	0.57	
Phosphotyrosine independent ligand for the Lck SH2 domain p62	0.65	
NF-kappaB inhibitor alpha-2	0.29	
Mitogen-activated protein kinase 13	-0.44	
NF-kappaB inhibitor alpha-3	0.53	
**Receptor binding (17)**		
Tumor necrosis factor receptor superfamily member 11B	0.78	0.33
Small inducible cytokine B14 precursor	0.34	
Alpha-taxilin	-0.89	
Proteinase activated receptor 1	0.90	
Glomulin	0.78	
Chemokine-like factor family member 3	0.58	
Melanoma derived growth regulatory protein	0.62	
B-cell receptor-associated protein 31	0.47	
Receptor for activated C kinase	0.35	
Heat shock protein 75 kDa-2	0.26	
Midkine precursor	0.32	
CC chemokine SCYA110-2	-0.38	
Laminin alpha-4 chain precursor	-0.71	
Glucose-6-phosphate isomerase-2	0.44	
B-cell receptor-associated protein BAP37-2	0.30	
T-cell receptor alpha chain V region HPB-MLT precursor (Fragment)	0.89	
Guanine nucleotide-binding protein beta subunit 2-like 1	0.60	
**Humoral immune response (17)**		
B-cell receptor CD22-1	0.44	0.36
GRB2-related adaptor protein 2	-0.16	
Cytochrome B-245 heavy chain-1	0.87	
Complement factor B/C2-B	1.08	
Tumor necrosis factor receptor superfamily member 5 precursor	0.75	
Complement component C9	0.51	
Mannan-binding lectin serine protease 2-1	-0.51	
Complement factor MASP-3	1.90	

Coatomer epsilon subunit 1	-0.42	
Serine protease-like protein-1	-0.44	
Adenosine deaminase 3	-0.35	
Serine protease-like protein-3	0.35	
CC chemokine SCYA110-2	-0.38	
Glucose-6-phosphate isomerase-2	0.44	
Transferrin	0.48	
Complement factor H-1	0.44	
Complement factor H-2	1.06	
**Cytoskeleton (18)**		
PDZ and LIM domain protein 1	0.52	0.31
Coronin-1C	-1.18	
Actin, cytoplasmic 2	0.52	
Beta actin-2	0.62	
Troponin T-3, fast skeletal muscle	0.35	
Chromosome-associated kinesin KIF4A	0.34	
Myristoylated alanine-rich protein kinase C substrate	0.34	
Thymosin beta-4-2	-0.28	
Myosin light chain 2-1	0.52	
Microtubule-associated protein RP/EB	-0.62	
Actin, alpha skeletal 2	0.34	
Actin, alpha skeletal 3	0.39	
Myosin heavy chain, skeletal, adult 1-1	0.41	
Tropomyosin alpha 3 chain-2	0.87	
Myosin heavy chain, cardiac muscle beta isoform	0.63	
Actin, alpha skeletal 4	0.59	
Myosin heavy chain, skeletal, adult 1-2	0.53	
Actin, alpha skeletal 5	0.69	
**Endopeptidase activity (18)**		
Cathepsin B-2	0.33	0.35
Cathepsin D-1	0.35	
Cathepsin Y	-0.34	
Complement factor B/C2-B	1.08	
Cathepsin D-2	0.53	
Mannan-binding lectin serine protease 2-1	-0.51	
Matrix metalloproteinase-2	0.29	
ADAMTS-3	0.40	
Complement factor MASP-3	1.90	
Tolloid-like protein (nephrosin)-1	0.76	
Serine protease-like protein-1	-0.44	
Cathepsin C-2	-0.31	
Serine protease-like protein-3	0.35	
Proteasome subunit alpha type 6	0.59	
Proteasome subunit alpha type 7-1	-0.53	
Transferrin	0.48	
Coagulation factor X precursor	0.36	
Plasminogen precursor-1	1.08	
**Plasma membrane (20)**		
Chemokine 5a receptor-like	0.73	0.35
C3a anaphylatoxin chemotactic receptor	-0.83	
B-cell receptor CD22-1	0.44	
Tumor necrosis factor receptor superfamily member 9	0.71	
Galectin-3	0.43	
Proteinase activated receptor 1	0.90	
Phosphatidylinositol 3,4,5-trisphosphate-dependent Rac exchanger 1	-0.28	
Tumor necrosis factor receptor superfamily member 5 precursor	0.75	

CD63	0.43	
Complement component C9	0.51	
Fibronectin receptor beta	0.74	
Regulator of G-protein signaling 1-1	0.23	
Vesicle-associated membrane protein-associated protein B/C	-1.15	
B-cell receptor-associated protein 31	0.47	
Tolloid-like protein (nephrosin)-1	0.76	
Ferritin heavy chain-1	0.41	
Na/K ATPase alpha subunit-1	0.21	
MIR-interacting saposin-like protein precursor	0.39	
T-cell receptor alpha chain V region HPB-MLT precursor (Fragment)	0.89	
Myristoylated alanine-rich protein kinase C substrate	0.34	
**Apoptosis (21)**		
Tumor necrosis factor receptor superfamily member 11B	0.78	0.28
NF-kappaB inhibitor alpha-1	0.48	
TNF decoy receptor	0.48	
Tumor necrosis factor receptor superfamily member 9	0.71	
NACHT-, LRR- and PYD-containing protein 2	0.43	
Receptor-interacting serine/threonine-protein kinase 2	0.44	
Proteinase activated receptor 1	0.90	
Voltage-dependent anion-selective channel protein 1	-0.30	
BCL2/adenovirus E1B 19-kDa protein-interacting protein 2	0.30	
Tumor necrosis factor receptor superfamily member 5 precursor	0.75	
Inhibitor of apoptosis protein 3	-0.88	
BCL2-associated athanogene 1	0.30	
Growth arrest and DNA-damage-inducible GADD45 beta	0.57	
Vasopressin-activated calcium-mobilizing receptor	-1.41	
TNF receptor associated factor 1	0.58	
Cold autoinflammatory syndrome 1 protein	0.45	
Ubiquitin ligase SIAH1	-0.27	
NF-kappaB inhibitor alpha-2	0.29	
B-cell receptor-associated protein 31	0.47	
NF-kappaB inhibitor alpha-3	0.53	
Nuclear protein 1	0.34	
**Structural constituent of ribosome (24)**		
60S ribosomal protein L5-1	-0.39	0.21
Ribosomal protein L6-1	0.80	
Mitochondrial ribosomal protein L4, isoform a	-0.46	
40S ribosomal protein S14 (PRO2640)	-0.18	
Ribosomal protein L11	-0.25	
60S ribosomal protein L7a-2	0.47	
60S ribosomal protein L10-1	0.28	
40S ribosomal protein S20	-0.60	
40S ribosomal protein S11	0.28	
60S ribosomal protein L36	-0.15	
Ribosomal protein S2	0.45	
40S ribosomal protein S15-2	0.40	
TGF beta-inducible nuclear protein 1	0.55	
Ubiquitin	0.34	
39S ribosomal protein L45, mitochondrial precursor	0.66	
28S ribosomal protein S16, mitochondrial precursor	0.67	
60S ribosomal protein L18	0.50	
60S ribosomal protein L32-2	0.22	
60S ribosomal protein L7	0.29	
40S ribosomal protein S3-1	0.33	
40S ribosomal protein S9-1	-0.23	

40S ribosomal protein S6	0.33	
Ribosomal protein L13	0.28	
Ribosomal protein L6-2	0.50	
**Protein biosynthesis (27)**		
Glomulin	0.78	0.23
Mitogen-activated protein kinase 13	-0.44	
Basic leucine-zipper protein BZAP45-1	0.57	
Alpha-2,8-sialyltransferase 8F	-0.42	
60S ribosomal protein L5-1	-0.39	
Ribosomal protein L6-1	0.80	
Mitochondrial ribosomal protein L4, isoform a	-0.46	
40S ribosomal protein S14 (PRO2640)	-0.18	
60S ribosomal protein L7a-2	0.47	
Eukaryotic translation elongation factor 1 alpha 1	0.45	
60S ribosomal protein L10-1	0.28	
40S ribosomal protein S20	-0.60	
40S ribosomal protein S11	0.28	
60S ribosomal protein L36	-0.15	
Ribosomal protein S2	0.45	
TGF beta-inducible nuclear protein 1	0.55	
Ubiquitin	0.34	
39S ribosomal protein L45, mitochondrial precursor	0.66	
Eukaryotic translation initiation factor 3 subunit 6-2	0.31	
28S ribosomal protein S16, mitochondrial precursor	0.67	
60S ribosomal protein L18	0.50	
60S ribosomal protein L7	0.29	
40S ribosomal protein S3-1	0.33	
40S ribosomal protein S9-1	-0.23	
Ribosomal protein L13	0.28	
Elongation factor 1-alpha 2	0.50	
Ribosomal protein L6-2	0.50	
